# Surface Defect Detection of Bearing Rings Based on an Improved YOLOv5 Network

**DOI:** 10.3390/s23177443

**Published:** 2023-08-26

**Authors:** Haitao Xu, Haipeng Pan, Junfeng Li

**Affiliations:** 1 School of Information Science and Engineering, Zhejiang Sci-Tech University, Hangzhou 310018, China; 18357660258@163.com (H.X.); ljf2003@zstu.edu.cn (J.L.); 2 Changshan Research Institute, Zhejiang Sci-Tech University, Quzhou 324299, China

**Keywords:** bearing ring, surface defect detection, deep learning, YOLOv5

## Abstract

Considering the characteristics of complex texture backgrounds, uneven brightness, varying defect sizes, and multiple defect types of the bearing surface images, a surface defect detection method for bearing rings is proposed based on improved YOLOv5. First, replacing the C3 module in the backbone network with a C2f module can effectively reduce the number of network parameters and computational complexity, thereby improving the speed and accuracy of the backbone network. Second, adding the SPD module into the backbone and neck networks enhances their ability to process low-resolution and small-object images. Next, replacing the nearest-neighbor upsampling with the lightweight and universal CARAFE operator fully utilizes feature semantic information, enriches contextual information, and reduces information loss during transmission, thereby effectively improving the model’s diversity and robustness. Finally, we constructed a dataset of bearing ring surface images collected from industrial sites and conducted numerous experiments based on this dataset. Experimental results show that the mean average precision (mAP) of the network is 97.3%, especially for dents and black spot defects, improved by 2.2% and 3.9%, respectively, and that the detection speed can reach 100 frames per second (FPS). Compared with mainstream surface defect detection algorithms, the proposed method shows significant improvements in both accuracy and detection time and can meet the requirements of industrial defect detection.

## 1. Introduction

As a component that plays a role in fixing and reducing the load friction in mechanical transmission processes, bearings are widely used to guide the rotational motion of shaft parts and withstand the loads transmitted by the shaft to the frame. The quality of the bearings in mechanical equipment has a significant impact on the stability of the entire equipment operation. However, during the production and assembly process of bearings, surface defects are inevitable due to factors such as materials, processing, assembly, and transportation. Common surface defect types include cracks, black spots, scratches, dents, forging waste, helical marks, undersized inner and outer ring chamfers, and incorrect character engraving, among others. These defects affect not only the appearance and quality of the bearings but also their service life and performance. Therefore, quality inspection of bearings must be carried out before they leave the factory.

In recent years, with the development of machine vision and deep learning technologies, many defect detection methods based on machine vision and deep learning have been widely applied in various industrial scenarios, mainly including solar energy [1,2], transportation [3,4], textile [5,6], medical [7,8,9], metal materials [10,11,12], and other fields. However, machine vision inspection methods for surface defects on bearings are not commonly utilized.

Currently, bearing surface defect detection methods can be roughly divided into two categories. One is traditional machine vision-based detection methods, Liu et al. [13] proposed an automatic synthetic tiny defect detection system for bearing surfaces with self-developed software and hardware, thresholding segmentation, contour extraction, contour filtering, center location, region zoning, and text recognition are successively implemented, but this method cannot detect small or too large defects. Jiang et al. [14] proposed a cloud model-improved EEMD with superior performance in reducing multiple background noise and then proposed a rolling bearing defect detection scheme based on this method, but this method will adversely affect the fault feature extraction. Li et al. [15] proposed a technique using the generalized synchrosqueezing transform (GST) guided by enhanced TF ridge extraction to detect the existence of the bearing defects. Wang et al. [16] proposed a synthetic detection technique that uses a combination of empirical mode decomposition (EMD), ensemble empirical mode decomposition (EEMD), and a deep belief network (DBN) to improve the accuracy of the acoustic defective bearing detector. The alternative is deep learning-based detection methods. Tabernik et al. [17] proposed a segmentation-based deep learning method for defect detection. The method first converts vibration signals into two-dimensional grayscale images in a time-frequency representation. Multiple binary segmentation models are then used to segment the images and obtain masks of the defect regions. Finally, a classification model is used to classify each defect region, which enables the detection and segmentation of surface cracks with a few defective samples. Xu et al. [18] proposed an unsupervised neural network approach based on autoencoder networks for bearing fault detection. The method preprocesses bearing images using a normalization algorithm that preserves the symmetry of the samples and uses gradients as labels to automatically detect surface defects on bearings. However, this method does not consider information such as the types, locations, or sizes of bearing defects but simply distinguishes defects from normal regions. Lei et al. [19] proposed a segmented embedded rapid surface defect detection (SERDD) method that achieves a bidirectional fusion of image processing and defect detection. The method uses the spatial pyramid character proportion matching (SPCPM) method for character recognition and the image self-stitching and cropping (ISSC) method to solve the problem of truncated characters during coordinate transformation and the recognition of imprinted characters on the dust covers of bearings. Li et al. [20] proposed a real-time steel strip surface defect detection method based on an improved YOLO detection network. The method improves the structure and loss function of the YOLO network and adds an attention mechanism and a multiscale feature fusion module. The mAP for six types of defects reached 97.55%, and the FPS reached 83. Fu et al. [21] proposed a two-stage attention-aware method based on convolutional neural networks for detecting oil leakage in bearings. They used a novel attention-aware network called APP-UNet16, which stacks attention to allow for adaptive changes in attention-aware features. However, this method requires precise positioning of the bearings. Kumar et al. [22] proposed a bearing defect size estimation method based on a wavelet transform and a deep convolutional neural network (DCNN). The method uses a continuous wavelet transform to process vibration signals and form two-dimensional grayscale images in a time-frequency representation. Then, the DCNN is used to learn the bearing defects and extract high-level features from the images, and the trained grayscale images are applied to the DCNN. However, this method relies on the selection of wavelet transform parameters, which may cause distortion and time-frequency information loss if the parameters are not properly chosen. Song et al. [23] proposed an object detection algorithm based on YOLOv4. The method modifies the loss function to a focal loss function to eliminate the problem of bearing background interference and uses a smooth activation function to improve the gradient descent optimization process.

The application prospects of object detection technology based on deep learning theory in the detection of surface defects in bearings are very promising, but there are still some difficulties. This is because the background texture of the bearing rings is complex, the sizes of the defects are different, the types are diverse, and the brightness is uneven. In addition, the production environment of bearings has a large amount of oil and dust, which can interfere with the bearing images. Based on this, in combination with the optical characteristics, imaging characteristics, and detection requirements of the bearing rings, an improved YOLOv5 defect detection network is proposed, and an automatic detection system for surface defects of bearings is developed, which is applied industrially. The primary contributions of this study are as follows:

1. The C3 module in the main network is replaced with a C2f module, which not only reduces the number of parameters and computational complexity but also yields features with higher levels of semantics and globality.
2. A new CNN module is constructed, and the SPD module is introduced into both the main and neck networks, thereby improving the ability to detect low-resolution and small-object images.
3. A lightweight and universal upsampling operator, CARAFE, is utilized to enrich contextual information, reduce information loss during transmission, and enhance both the defect detection capability and the diversity and robustness of the network.

The remainder of this paper is organized as follows. Section 2 introduces the bearing defect detection system. Section 3 presents the detection method in detail, including details network structure of the improved YOLOv5 and loss function. Section 4 provides experimental validation of our method. Finally, Section 5 is the conclusion of our work.

## 2. Bearing Defect Detection System

### 2.1. Bearing Defect Detection Device

**Figure 1:**
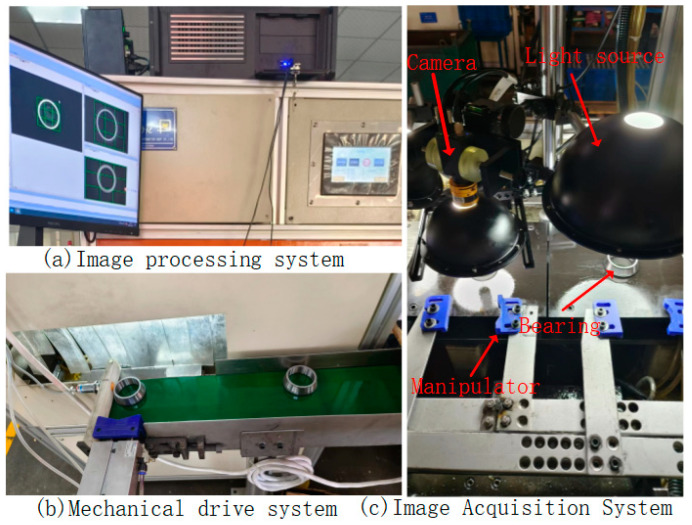
Bearing defect detection device.

The bearing defect visual inspection device designed and developed in this study, as shown in Figure 1, consists of three main parts: mechanical transmission, machine vision, and electrical control. The mechanical transmission section is composed of a frame, motor, belt, and cylinder, which transports the bearings to various inspection stations. The machine vision section is composed of an industrial camera, vision light source, industrial computer, and inspection system software, which provides high-quality imaging of the transported bearings and achieves the precise and real-time detection of various defects. The electrical control section is composed of a PLC and photoelectric sensors, which trigger the industrial camera and vision light source and reject defective products. Table 1 shows the system equipment models.

### 2.2. Bearing Defect Type

The formation of bearing defects occurs mainly during raw material processing, loading and unloading, transportation, and measurement. The defects include mainly helical marks, forging waste, black spots, dents, and scratches. In the forging process, temperature differences can cause forging waste to form. During the turning process, slight displacement due to high-speed machine operation can result in excessive cutting and cause helical marks or scratches. In the rust prevention process, uneven application of rust-preventive oil and the humid production environment can cause rust and black spots to form on the bearings. During transportation, collisions on the surfaces of the bearings can cause dents and scratches.

(1) Helical marks

**Figure 2:**
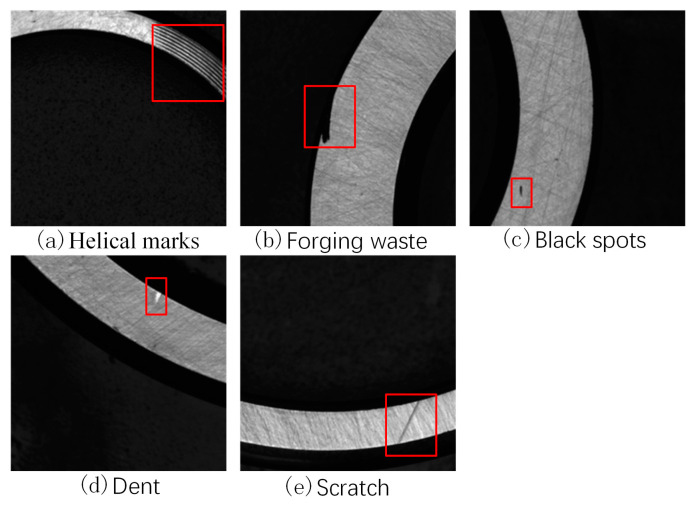
Images of different types of defects in bearings.

A helical mark defect, shown in the red box of Figure 2a, appears as a spiral-shaped thread on the outer diameter of the roller, resembling knife-like scratches caused by sharp burrs and edges on the grinding wheel, guide wheel, and cutting plate. The color is usually black, with a size of 0.5 × 1.0 mm or more.

(2) Forging waste

A forging waste defect, shown in the red box of Figure 2b, is an unground portion left during the grinding process. The color is usually black, with a size of 1.0 × 1.0 mm or more.

(3) Black spots

A black spot defect, shown in the red box of Figure 2c, is a discolored pitting caused by chemical etching on the surface, usually appearing as dark spots. The color is usually black, with a size of 0.3 × 0.3 mm or more.

(4) Dents

A dent defect, shown in the red box of Figure 2d, is caused mainly by local sinking and protrusion on the surfaces of the bearing parts due to collisions during the production, loading, unloading, and transportation processes. The color is usually white, with a size of 0.5 × 0.5 mm or more.

(5) Scratches

A scratch defect, shown in the red box of Figure 2e, is a surface defect with a certain depth that appears as a linear scratch and is mainly caused by the processing, loading, unloading, and measurement of the workpiece. The color is usually black, with a size of 0.1 × 0.1 mm or more.

## 3. Bearing Rings Defect Detection Model Based on the Improved YOLOv5

### 3.1. Network Structure of the Improved YOLOv5

YOLOv5 employs CSPDarknet53 as the backbone network and combines a feature pyramid network (FPN) and path aggregation network (PAN) as the neck network to fuse the features extracted from the backbone [24]. The main part of the output head consists of three Detect detectors, which perform object detection using grid-based anchors on feature maps of different scales.

**Figure 3:**
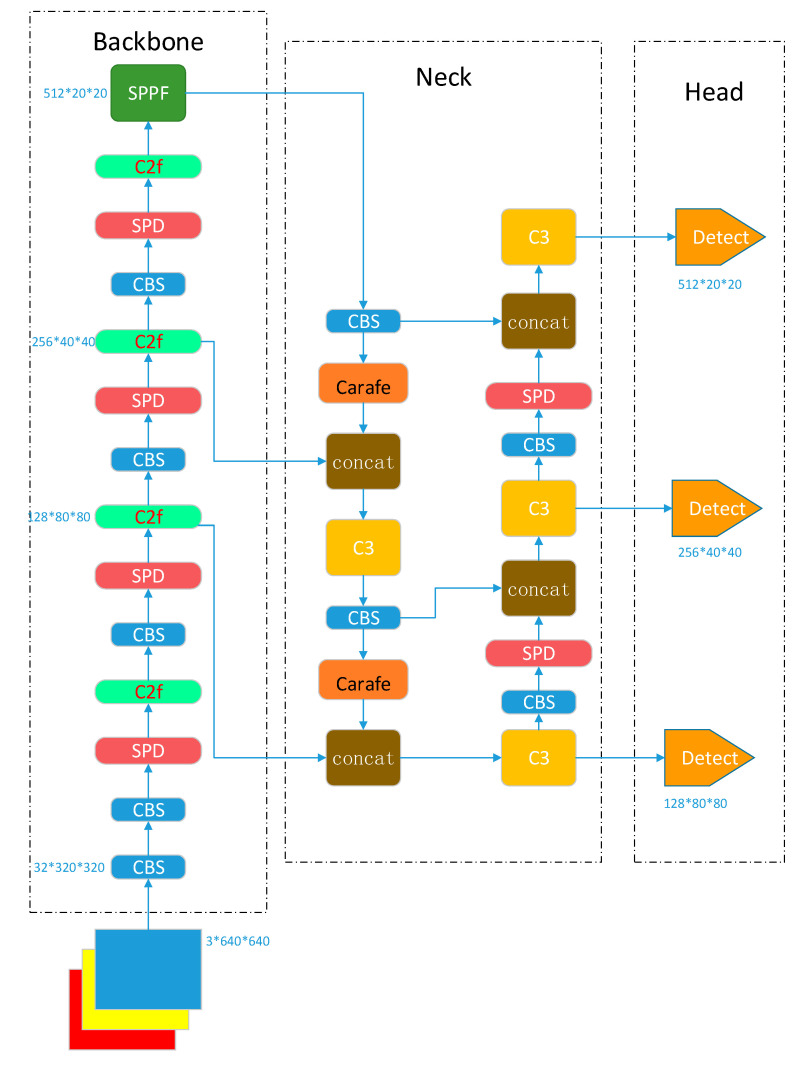
Network structure of the improved YOLOv5.

In the YOLOv5 network, strided convolutions and pooling layers can lead to the loss of fine-grained information and a decrease in feature extraction ability. The parameter and computational complexities of the C3 module, which affect the detection speed, are relatively high. Moreover, in the neck network, the use of the nearest-neighbor interpolation algorithm for feature map sampling can result in obvious jaggedness, leading to missing feature details and structural information in the feature map. Bearing defects such as helical marks, forging waste, black spots, dents, and scratches have different sizes and shapes, requiring the effective fusion of shallow and high-level semantic information across different scales of features. Based on this, to achieve the high-precision and high-efficiency detection of bearing defects on the surfaces of bearing races in complex scenes, this paper proposes an improved YOLOv5 network, as shown in Figure 3. The network consists of three parts: the backbone, neck, and head. The backbone is used for feature extraction, the neck is used for the multiscale fusion of features extracted from different levels of the backbone using both top-down and bottom-up approaches, and the output is used for object detection and classification.

The backbone network performs five downsampling operations on the input through convolution, batch normalization, and SiLU activation function-based CBS downsampling modules.

In this paper, we use the SPD module to replace the previous Conv layer for downsampling, which increases the number of channels in the feature maps. The ability to detect low-resolution images and small objects is improved while maintaining the resolution of the feature map, which improves the model expression and generalization ability. Additionally, a C2f module is utilized to extract features. In contrast to the C3 module, C2f uses separable convolution and concatenation operations to enable the fusion of feature maps with different numbers of channels. In the neck, we employ CARAFE for upsampling, which preserves more detailed feature information and structural information, thereby improving the quality and accuracy of the upsampling operation.

The backbone network is a convolutional neural network that extracts features of different sizes from the input image through multiple convolutions and SPD modules. The input image size is 640 × 640 pixels, and the backbone network generates five layers of feature maps after 2, 4, 8, 16, and 32 downsampling operations. The sizes of these feature maps are 320 × 320, 160 × 160, 80 × 80, 40 × 40, and 20 × 20, respectively.

To obtain more contextual information and reduce information loss during transmission, the neck network fuses the feature maps of the third, fourth, and fifth layers of the backbone network to enhance the feature fusion capability of the neck network. During the fusion process, the FPN structure transmits shallow semantic information from the top down, while the PAN structure transmits deep semantic information from the bottom up. These two structures jointly enhance the feature fusion capability of the neck network, and after feature fusion, three new feature maps are generated through three output layers. These three output layers are the shallow, middle, and deep layers, and the output sizes are 80 × 80 × 128, 40 × 40 × 256, and 20 × 20 × 512, respectively, where 128, 256, and 512 represent the numbers of channels. The smaller a feature map is, the larger the image area represented by each grid cell in the feature map. The shallow feature map is suitable for detecting small targets, the middle feature map is suitable for detecting medium targets, and the deep feature map is suitable for detecting large targets. Based on these new feature maps, the output network performs object detection and classification.

### 3.2. Space-to-Depth

In the task of detecting surface defects on bearings with low image resolution or small objects, the performance of convolutional neural networks for computer vision tasks such as image classification and object detection will rapidly degrade. The reason for this is that existing CNN architectures use strided convolutions and pooling layers, which lead to the loss of fine-grained information and the learning of less-efficient feature representations.

The space-to-depth (SPD) [25] module can solve the problems of information loss and performance degradation caused by traditional strided convolutions or pooling layers when processing low-resolution images and small objects. The SPD module downsamples the feature map (X) within the entire network while retaining all the information in the channel dimension without information loss. A non-strided convolution layer is added after each SPD layer, which uses learnable parameters in the increased convolutional layer to reduce the number of channels and reduce the non-discriminatory loss of information. In this study, we use the SPD module for downsampling and change the stride of the Conv layer in the layer above the SPD module from 2 to 1.

The original image or intermediate feature map is split into a series of sub-feature maps by the SPD layer, which are then stacked together, thereby increasing the number of channels and enlarging the receptive field while reducing the spatial dimensions. For any intermediate feature map *X* of size *S* × *S* × *C*, the series of sub-feature maps that are cut out are

(1) $$f_{\left(0,0\right)}=X[0:S:scale,0:S:scale]$$

(2) $$f_{\left(scale-1,0\right)}=X[\mathrm{scale}-1:S:scale,0:S:scale]$$

(3) $$f_{\left(0,scale-1\right)}=X[0:S:scale,scale-1:S:scale]$$

(4) $$f_{\left(scale-1,scale-1\right)}=X[scale-1:S:scale,scale-1:S:scale]$$

**Figure 4:**
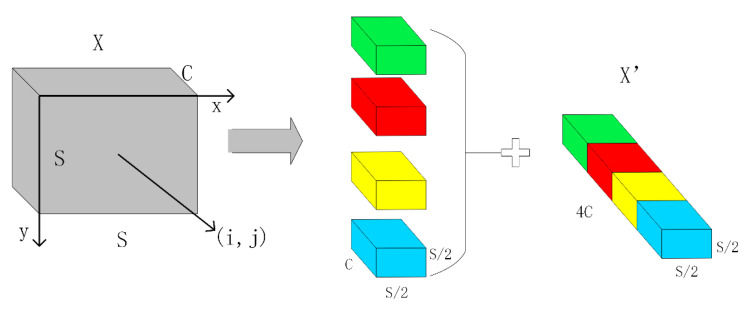
SPD schematic diagram.

In general, given any original feature map *X*, a sub-map f(x,y) is formed by all the entries  X(i, j) that (i+x) and (j+y) are divisible by scale. Therefore, each sub-map downsamples *X* by a factor of scale. When scale = 2, as shown in Figure 4, four sub-feature maps are obtained, each with a size of (S/2, S/2, C). Meanwhile, *X* is downsampled by a factor of 2, and the resulting sub-feature maps are concatenated along the channel dimension, resulting in a new feature map X’. The spatial dimensions of X’ are one-fourth of *X*, while its channel dimension is four times that of *X*.

The SPD module can reduce the computational complexity by using an SPD layer instead of strided convolutional or pooling layers. The SPD layer reduces the height and width of the input feature map by half while increasing the number of channels by four times, thereby increasing the depth of the feature map and maintaining the total number of elements, which improves the representation ability and multiscale fusion performance of the feature map. As a result, subsequent C2f layers can perform computations on a smaller spatial scale without losing information.

The SPD module can improve the receptive field and localization accuracy by preserving all information of the input feature map without losing details, such as strided convolutional or pooling layers. The SPD module can enhance the model performance, particularly in handling more challenging tasks such as low-resolution images and small objects.

### 3.3. C3 Module and C2f Module

#### 3.3.1. C3 Module

**Figure 5:**
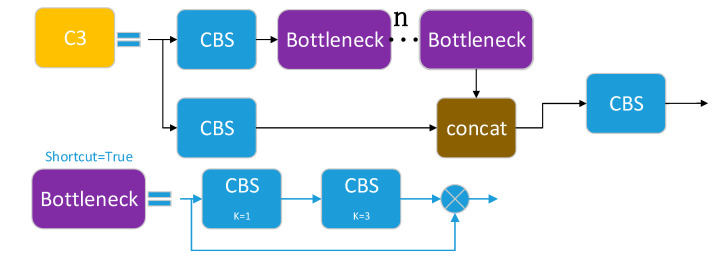
Structure diagram of C3.

The C3 module consists of three standard convolutional layers and n bottleneck modules, where the value of n depends on the model’s scale. It is the primary module for learning residual features, and its function is to increase the number of channels of the feature map while maintaining its size, thereby improving the feature representation performance. The bottleneck modules in the backbone use shortcuts, while those in the neck do not. As shown in Figure 5, the input feature map enters two branches, one of which generates a map, namely, sub-feature map 1, by stacking multiple bottleneck modules and one standard convolutional layer, while the other generates another map, namely, sub-feature map 2, with only one basic convolutional module. Finally, the two sub-feature maps are concatenated and output.

#### 3.3.2. C2f Module

The C2f module [26] was designed based on ideas from both the C3 module and ELAN. It utilizes one fewer CBS convolution than the C3 module and concatenates all the sub-feature maps output by each bottleneck module, which not only ensures a lightweight but also produces richer gradient flow information. Feature segmentation and fusion are achieved using the Split and Concat operations, respectively. Similar to the C3 module, the bottleneck modules in the backbone network use shortcut connections, while those in the neck network do not.

**Figure 6:**
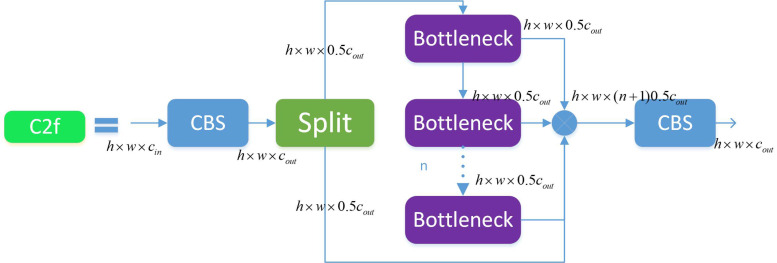
Structure diagram of C2f.

According to Figure 6, the size of the input feature map is $h\times w\times c_{in}$. After passing through the CBS, the output size is $h\times w\times c_{out}$. The output feature map is split into two sub-feature maps through the Split operation, and one sub-feature map is processed through n bottleneck modules. Meanwhile, each sub-feature map output by the bottleneck module is concatenated with a sub-feature map previously split by the Split operation. Finally, a CBS module is used to output a feature map with the same size as the input.

In the network architecture, the C2f module does not use shortcut connections by default, while the C3 module uses shortcut connections by default. Compared with the C3 module, the C2f module is of lighter weight and uses fewer parameters and computational resources while maintaining high accuracy and speed. Its function is to extract feature maps in the backbone and achieve feature fusion and channel separation through the CSP structure, improving the quality and efficiency of the feature maps, enhancing the receptive field and multiscale ability of the feature maps, and obtaining more global and higher-level semantic features.

### 3.4. Lightweight Universal Upsampling Operator

#### 3.4.1. Nearest-Neighbor Interpolation

The purpose of an upsampling module is to expand a low-resolution image or feature map into a high-resolution image or feature map, which can be displayed on higher-resolution display devices or improve the performance of subsequent tasks. An upsampling module can be used as an intermediate layer in a convolutional network to expand the size of the feature map and facilitate tensor concatenation. There are various methods for implementing upsampling, such as nearest-neighbor interpolation, bilinear interpolation, bicubic interpolation, trilinear interpolation, deconvolution, and transpose convolution [27]. Upsampling modules are commonly used in tasks such as image segmentation, super-resolution, and style transfer. Almost all upsampling methods use interpolation, i.e., inserting new elements between pixel points using appropriate interpolation algorithms based on existing image pixels.

**Figure 7:**
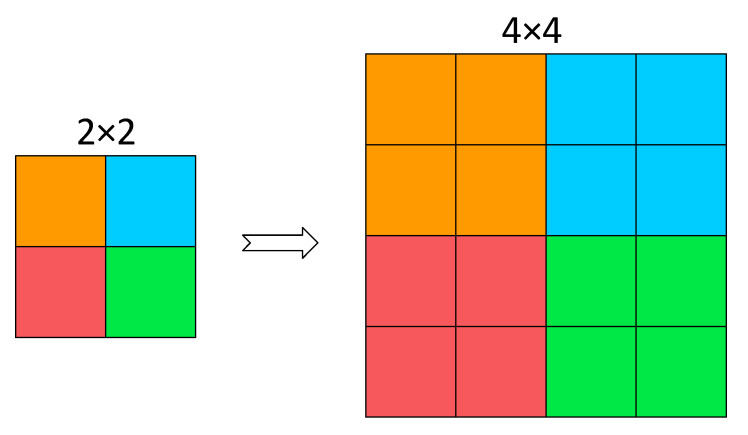
Nearest-neighbor interpolation.

In YOLOv5, nearest-neighbor interpolation is used as the default upsampling algorithm. Nearest-neighbor interpolation is the simplest interpolation algorithm, which sets the grayscale value of the transformed pixel to the grayscale value of the input pixel that is closest to it. Nearest-neighbor interpolation is implemented through coordinate transformation, mapping each pixel point in the target image to the original image and then taking the grayscale value of the original image pixel nearest to the target image pixel as the grayscale value of the target image pixel, as shown in Figure 7. When an image is enlarged, each missing pixel is generated directly using the nearest existing color, which means copying the neighboring pixels. However, this method produces obvious jaggedness. Jaggedness, commonly referred to as “jagged artifacts”, is a common phenomenon in image processing and computer graphics. They typically occur when resizing images or performing pixel-level resampling. The primary cause of this distortion is insufficient image resolution or inadequate smoothing of image details during image upscaling or resizing. When an image contains sharp transitions or edges between regions, low-quality interpolation methods, such as nearest-neighbor interpolation, can lead to abrupt changes in pixel values. This can result in pronounced jagged edges between edges and regions.

#### 3.4.2. CARAFE

CARAFE stands for content-aware reassembly of features [28], which is a lightweight and universal upsampling operator that can guide the upsampling process based on the semantic information of the input feature map. Its main strategy is to use a small convolutional network to generate an adaptive upsampling kernel and then to calculate the dot product of the kernel with the corresponding neighboring pixels in the input feature map to obtain the upsampled feature map. Compared with traditional upsampling operators such as nearest-neighbor or bilinear interpolation, CARAFE has a larger receptive field and better semantic adaptability while introducing very few parameters and not substantially increasing the computational cost.

**Figure 8:**
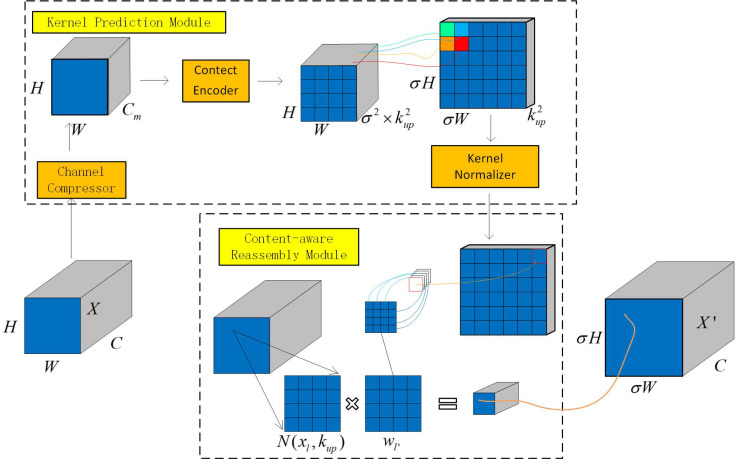
CARAFE schematic diagram.

The network structure of CARAFE consists of two parts, as shown in Figure 8. One part is the kernel prediction module, which is used to generate weights for the kernel used in the reassembly calculation. The other part is the content-aware reassembly module, which is used to reassemble the features with the calculated weights.

In Figure 8, a feature map X of size C×H×W is upsampled σ times using CARAFE. For each position l=(i,j), a kernel is predicted for recombination. First, the channel compression module compresses the channel to $C_{m}$ to reduce subsequent computation and enable the use of larger kernels during upsampling. Then, based on the size of the compressed feature map, a convolutional layer of size $k_{encoder}$ is used to generate the kernel for feature recombination, where using a larger $k_{encoder}$ expands the receptive field, while the channel dimensions become $\sigma^{2}\times k_{up}^{2}$. The new feature map is then recombined into the feature map of size $k_{up}^{2}\times \sigma H\times \sigma W$, and the softmax function is applied to normalize all channels at each position.

(5) $$W_{l\prime }=\mu (N(X_{l},k_{encoder})$$

(6) $$X_{l}\prime =\phi (N(X_{l},k_{up}^{}),W_{l\prime })$$

For any position in the output *X’*, there is a corresponding source position l=(i,j) in the input *X*, where $i=\left(\frac{i^{\prime }}{\sigma }\right),j=\left(\frac{j^{\prime }}{\sigma }\right)$. We let $N(X_{l},k_{up})$ be a $k_{up}\times k_{up}$ subregion of *X* centered at position l. The predicted kernel module μ predicts the position kernel $W_{l\prime }$ for each position l′ based on a subregion of $X_{l}$, as shown in Equation (5). In Equation (6), the perception recombination module ∅ recombines the subregion of $X_{l}$ with the position kernel $W_{l\prime }$ to obtain $X_{l}\prime$.

In the YOLOv5 network architecture, the introduction of CARAFE enables the dynamic generation of different upsampling kernels at different positions of the input feature map, adapting to targets of different scales and shapes in various instances and scenarios. The resulting upscaled feature map, obtained by calculating the inner product with the local neighborhood of the input feature map, possesses higher resolution and richer detail information, thereby enhancing the ability to recognize and locate different targets in object detection tasks. At the same time, with the introduction of only a small number of parameters and a low computational cost, compared to other upsampling methods, such as nearest-neighbor interpolation and deconvolution, the model has a smaller size and faster running speed, thereby meeting the real-time and efficiency requirements of object detection tasks.

### 3.5. Loss Function

A loss function is a function used to calculate the difference between predicted values and true values. The smaller the value of the loss function is, the closer the predicted output is to the expected output [29]. In this study, the applied loss function is divided into three parts: classification loss, localization loss, and confidence loss. The classification loss is used to determine whether the anchor box and corresponding annotated classification are correct and represents the probability of belonging to a certain category. The localization loss is used to predict the error between the predicted box and the annotated box. The confidence loss is used to calculate the network’s confidence. It represents the probability of there being an object and typically has a value between 0 and 1, with larger values indicating a higher probability of there being an object. The overall loss function is a weighted sum of these three loss functions, as expressed in Equation (7):

(7) $$LOSS=w_{box}L_{box}+w_{obj}L_{obj}+w_{cls}L_{cls}$$

where $w_{box}$, $w_{obj}$, and $w_{cls}$ are weighting coefficients.

The localization loss $L_{box}$ is defined using CIOU as [30]

(8) $$L_{box}=\mathrm{CIOU}=1-IOU+\frac{\rho^{2}(A,B)}{c^{2}}+\alpha \nu$$

where IOU is the intersection over union between the predicted box and the ground-truth box, with a larger value indicating a closer match; ρ represents the Euclidean distance between the center-point coordinates of the ground-truth box A and the predicted box B; c represents the diagonal distance of the minimum bounding box enclosing both the predicted box and ground-truth box; α is a weighting coefficient; and ν is used to measure the consistency between the aspect ratios of A and B.

IOU is defined as

(9) $$IOU=\frac{A\cap B}{A\cup B}$$

where A is the ground-truth bounding box, B is the predicted bounding box, A∩B represents the intersection of A and B, and A∪B represents the union of A and B.

α and ν are defined as follows:

(10) $$\alpha =\frac{\nu }{1-IOU+\nu }$$

(11) $$\nu =\frac{4}{\pi^{2}}{\left(\arctan\frac{w^{B}}{h^{B}}-\arctan\frac{w}{h}\right)}^{2}$$

The binary cross-entropy with the logit loss is used for both the classification and confidence losses in this study, which is defined as follows:

(12) $$L_{cls}=L_{obj}=-\frac{1}{n}\sum_{i=1}^{n}\left(y_{i}\times \ln x_{i}+\left(1-y_{i}\right)\times \ln\left(1-x_{i}\right)\right)$$

where n represents the number of input samples, $y_{i}$ represents the target values, and $x_{i}$ represents the predicted output values.

## 4. Experimental Verification

### 4.1. Bearing Surface Defect Dataset

**Figure 9:**
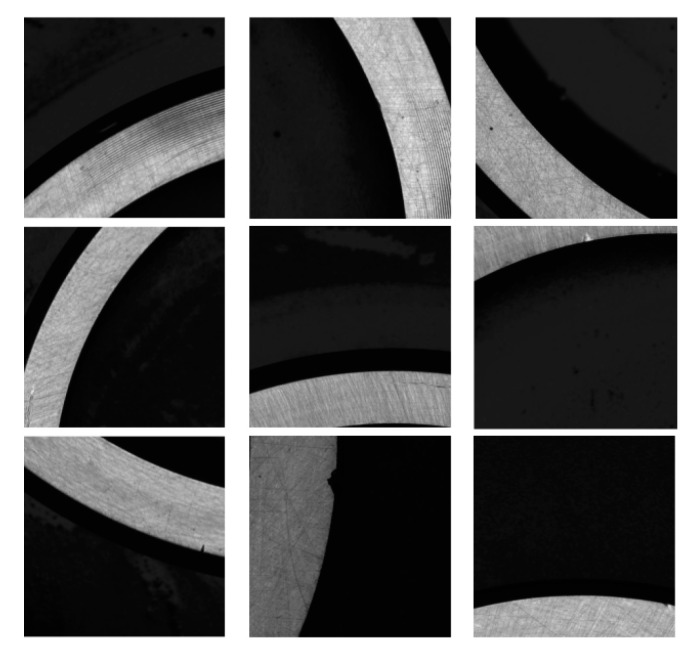
Dataset of surface defects of various bearing rings.

The bearing rings defect dataset used in this study was collected from the industrial field and captured by cameras on a factory production line. The captured images had a resolution of 5472 × 3468, and each image was approximately 19 MB in size. Images containing defects were manually cropped into windows of size 640 × 640. Defective images were selected, and the dataset was categorized based on defect type, including helical marks, forging waste, black spots, dents, and scratches. The dataset we constructed is shown in Figure 9. Due to differences in the actual numbers of defects in production, to ensure training rationality and balance among the defect types, the number of defects was augmented to 5660. The statistical data for each type of defect after augmentation are shown in Table 2.

Before training the network with the dataset, it was necessary to divide the dataset into training, validation, and testing sets based on the sample size and training rationality. In this study, each type of defect sample was divided into training, validation, and testing sets at a ratio of 6:2:2. The results of the dataset division are shown in Table 3.

### 4.2. Experimental Setup and Data Enhancement

#### 4.2.1. Experimental Setup

The hardware and software versions used in the experiments are shown in Table 4.

The training parameters of the network can also affect the performance of the model. The parameters used in this study are specified in Table 5.

#### 4.2.2. Data Augmentation

To enrich the information on the detected targets and improve the robustness of the network, data augmentation methods were used during the training process. GridMask [31] randomly generates a grid-shaped occlusion on an image, with a pixel value of 0 inside the occlusion, while the classification results remain unchanged. However, this method may reduce the clarity and quality of the image. RandAugment [32] is an automatic data augmentation method that randomly selects two transformations from a predefined set and applies them to an image with a random magnitude. However, it may introduce some transformations that are too strong or unsuitable, such as color distortion or object deformation, which can reduce the recognizability of the image.

**Figure 10:**
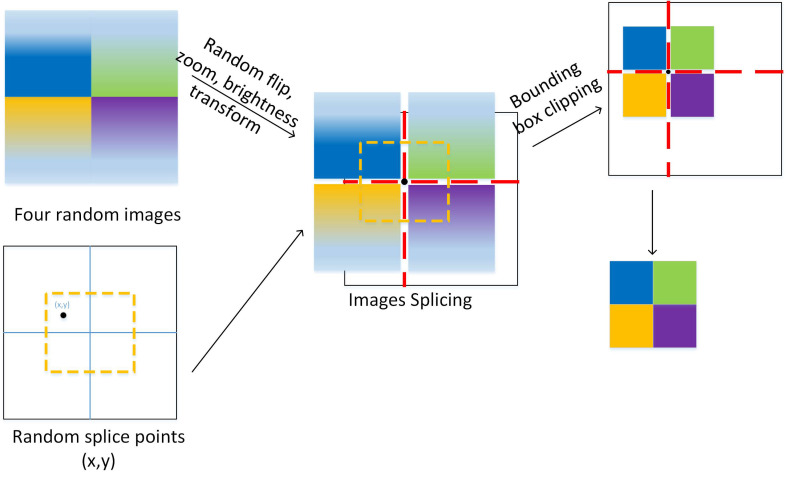
Mosaic data augmentation.

Mosaic data augmentation [33] is a method of combining four images into one by selecting four images, resizing them to the same size, randomly selecting a cutting point, cutting each image into four parts, and then combining different parts of different images into a new image while preserving the labels of the original images. Finally, other data augmentation operations, such as random rotation, cropping, scaling, and brightness adjustment, are applied to the new image. The principle of this method is shown in Figure 10. Mosaic data augmentation can improve the performance of object detection tasks, especially for small objects and dense scenes, by enriching the dataset of small targets. It can also increase the diversity and complexity of the training images, thereby improving the generalization ability of the model.

### 4.3. Performance Indices

To evaluate the performance of the improved YOLOv5 defect detection model, the mAP, average precision (AP), and FPS were evaluated [34]. The confusion matrix is shown in Table 6.

The matrix terms are defined formally as follows:

True Positive (TP): The model correctly predicted a positive-class (defect) sample as positive.

False Positive (FP): The model incorrectly predicted a negative-class (non-defect) sample as positive.

False Negative (FN): The model incorrectly predicted a positive-class (defect) sample as negative.

True Negative (TN): The model correctly predicted a negative-class (non-defect) sample as negative.

The number of images that the object detection network can detect per second is represented by FPS. The larger FPS is, the more images the object detection network can process per second and the faster the processing speed.

The calculation formulas for accuracy and recall rate are as follows:

(13) $$P=\frac{\mathrm{TP}}{\mathrm{TP}+\mathrm{FP}}$$

(14) $$R=\frac{\mathrm{TP}}{\mathrm{TP}+\mathrm{FN}}$$

AP and mAP are defined as follows:

(15) $$\mathrm{AP}=\int_{0}^{1}P\left(R\right)dR$$

(16) $$m\mathrm{AP}=\frac{\sum_{n=0}^{c}\mathrm{AP}\left(C\right)}{C}$$

The AP is the area under the precision-recall (P-R) curve. The mAP represents the average AP value for each category and is used to measure the detection performance of the network model for all categories.

### 4.4. Hyperparametric Study

Different training parameters can affect the performance of a deep learning model, including the input image size, number of training epochs, batch size, learning rate, and optimizer. In this study, the parameters used for training were those listed under exp1 in Table 7. To determine whether these parameters were optimal, multiple experiments were conducted by adjusting them, and the performance of the improved YOLOv5 on the bearing surface defect dataset was evaluated. The experimental results are shown in Table 7.

During the experiments, it was observed that the change in the loss function became stable when the number of training epochs approached 100. Therefore, in this study, the number of training epochs was set to 100. Moreover, as shown in Table 7, exp1 had the highest mAP value among the tested parameter settings, which validated the rationality of the parameter settings used in this study. In contrast, exp6 had the lowest mAP value, indicating that the choices of batch size and optimizer significantly impacted the experimental results.

### 4.5. Ablation Experiment

This study made three improvements to YOLOv5. To verify the effectiveness of each improvement and the combined effectiveness of each pair of improvements, ablation experiments were conducted, and the results are shown in Table 8. The ‘✓’ indicates the presence of improvement.

As shown in Table 8, the mAP of YOLOv5 is 95.8%, and the mAP of adding the C2f module is 96.5%. At the same time, the parameter quantity has been reduced by 91,488, FPS increased by 5, the C2f module compared to the C3 module in terms of decreasing parameter count while significantly enhancing the speed of bearing surface defect detection. With the addition of the CARAFE module, the mAP was 96.2%; With the addition of the SPD module, the mAP was 96.5%. When both the C2f and CARAFE modules were used, the mAP increased to 96.9%, indicating that the combination of these two improvements also helps improve the detection of bearing surface defects. The mAP further increased to 97.3% when all three modules were combined, which not only improved the feature extraction performance of the backbone network but also enhanced the quality and accuracy of upsampling and reduced the number of parameters. By fusing more semantic information into the pyramid layer during the feature fusion stage, more feature details and structural information were retained, which improved the detection capability for low-resolution images and small objects.

### 4.6. Experimental Results and Comparison

#### 4.6.1. Experimental Results on the Bearing Surface Defect Dataset

**Figure 11:**
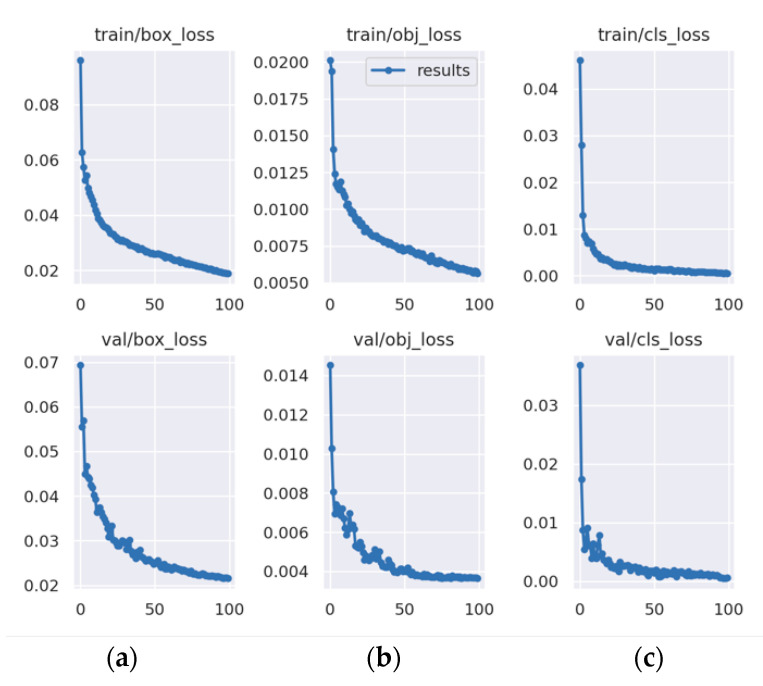
Training loss and validation loss. (**a**) Localization loss; (**b**) confidence loss; and (**c**) classification loss.

**Figure 12:**
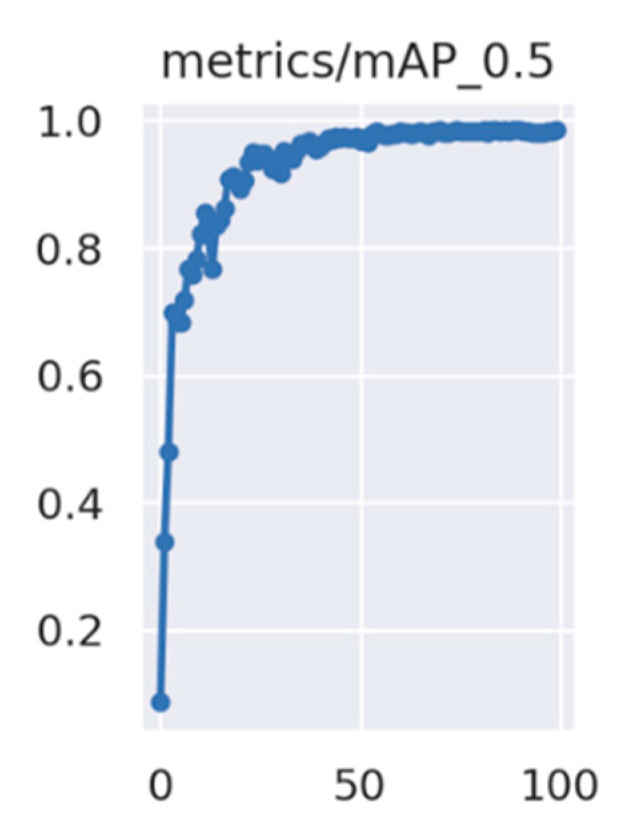
mAP curve.

**Figure 13:**
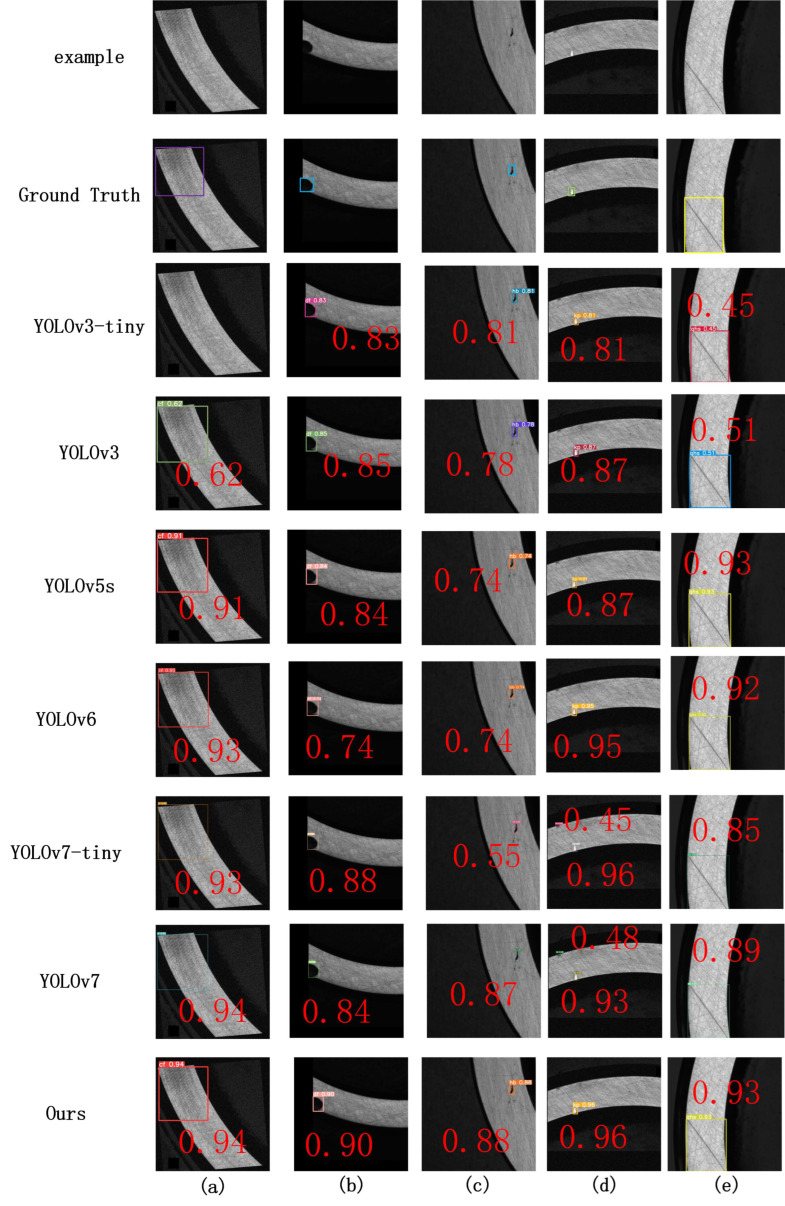
Test results on the bearing defect dataset for different models. (**a**) Helical marks; (**b**) forging waste; (**c**) black spots; (**d**) dents; and (**e**) scratches.

To further validate the effectiveness of the improved YOLOv5 defect detection model, this study compared it with object detection methods such as SSD, Faster-RCNN, YOLOv3, YOLOv5, YOLOv6, and YOLOv7. The training loss, validation loss, and mAP curves during training are shown in Figure 11 and Figure 12, and the comparison results for each model are shown in Figure 13. The experimental results are summarized in Table 9.

Figure 11 shows that the training and validation loss converged quickly within the first 30 training epochs and converged completely when the number of training epochs reached 100. Figure 12 shows that the mAP increased as the number of training epochs increased.

Table 9 shows that the improved YOLOv5 defect detection method proposed in this study outperformed other object detection methods. The mean average precision of the Faster-RCNN model was the lowest, at 83.6%, which does not meet the detection requirements. The mean average precision of the YOLOv5 model was 95.8%, while the mean average precision of the improved YOLOv5 proposed in this study was 97.3%, representing an overall increase in accuracy of 1.5%. Specifically, the accuracy of detecting black spots improved by 3.9%, while the accuracy of detecting dents improved by 2.2%, representing significant improvements. There was a slight decrease in FPS.

In this study, five images were randomly selected for testing on various models, and the results are shown in Figure 13. Different models had different detection performances on the bearing defect dataset. Among them, the SSD model fails to detect dents defects. The Faster-RCNN model fails to detect helix marks and dents defects, validating the low precision of the Faster-RCNN model. Obviously, our model has better detection performance compared with other detection models.

#### 4.6.2. Reasons for Misdetection

Three reasons for false detection are as follows:

(1) Issues with manual labeling

Because the bearing surface defect dataset originated from high-quality images collected from industrial sites, the 5660 images in the dataset had to be manually labeled. The amount of labeling required was huge, time-consuming, and inevitably prone to labeling errors.

(2) Existence of similar defects among different types of defects

The bearing defect dataset contained a variety of defect types, such as slightly larger black spot defects that were somewhat similar in shape to forging waste defects. In addition, concave defects may not be recognized under high brightness, and forging waste defects may be missed under low brightness. Some types of defects are difficult to accurately distinguish from others.

(3) Interference in the workshop environment

The bearing defect dataset was collected in an industrial site where the workshop environment may have caused interference. The bearings may have had oil stains, dust, and other factors that affected the accuracy of defect recognition. Some oil stains have shapes that are similar to some types of defects, which can interfere with accurate defect detection.

**Figure 14:**
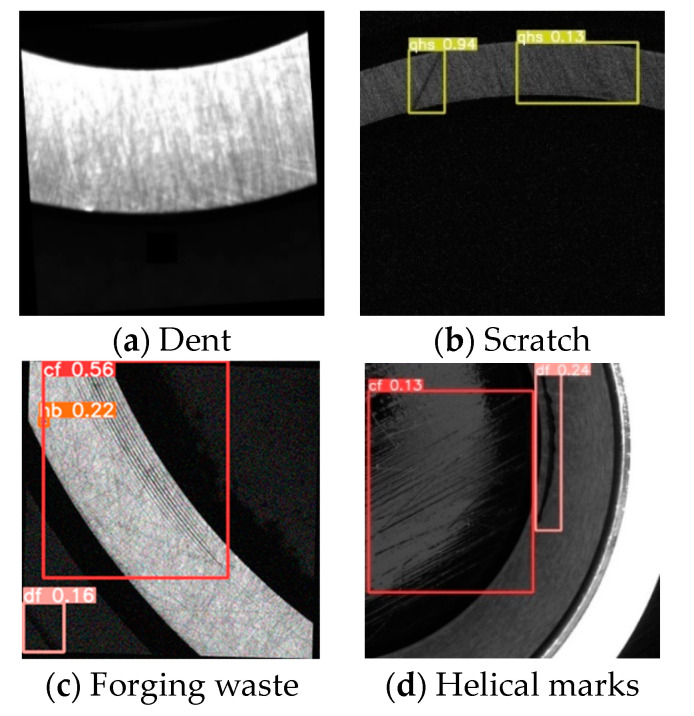
False detection and missed detection maps.

False detection and missed detection examples are shown in Figure 14.

Figure 13 (a) represents a missed detection of a dent defect caused by the small size of the defect, which was relatively rare in the dataset and has a silver-white color that is difficult to detect under high exposure; (b) on the right side is the normal area of the bearing, but the prediction result shows a scratch defect; In (c), a forging waste defect was falsely detected, while in (d), a helical mark defect was falsely detected.

### 4.7. Experimental Results for Fabric Defect Detection

To further evaluate the performance of the improved algorithm proposed in this study, we conducted comparative experiments on a fabric dataset. Following the experimental method used to detect surface defects on bearings, we first augmented a collected fabric dataset. Each fabric image was of size 400 × 400, and there were a total of 878 images. The dataset was expanded to 3317 images by applying horizontal flipping, brightness variation, and other methods. Then, the dataset was divided into training, validation, and testing sets at a ratio of 6:2:2. A comparison with YOLOv5 is shown in Figure 14, and a comparison with other algorithms is shown in Table 10.

**Figure 15:**
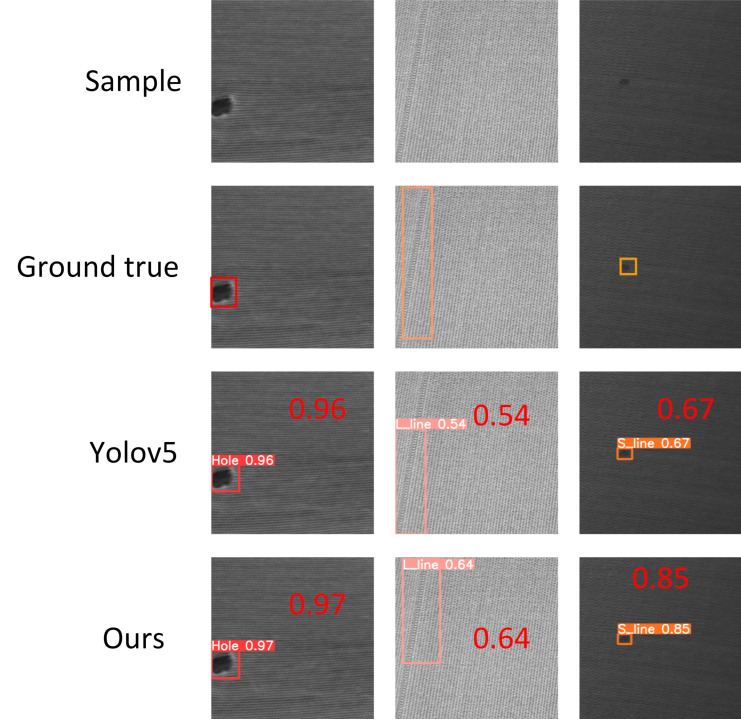
Comparative experimental results on the cloth surface defect dataset.

The results in Figure 15 demonstrate that the improved YOLOv5 algorithm outperformed the YOLOv5 model. As shown in Table 9, consistent with the experimental results on the bearing surface defect dataset, the improved YOLOv5 method consistently showed the best results, with the mAP of 99%. These results indicate that the proposed improved YOLOv5 model is effective and practical.

## 5. Conclusions

In response to the complex and varied characteristics of the bearing surface image texture background, uneven brightness, and defects of different sizes and types, this paper proposes a bearing surface defect detection method based on the improved YOLOv5. To improve the speed and accuracy of the YOLOv5 backbone network, the C3 module in the backbone network was replaced with a C2f module, reducing the number of parameters and computational complexity. To enhance the ability to process low-resolution and small-bearing images, the SPD module was added to the backbone network and neck network, and a new CNN module was constructed. To improve the diversity and robustness of the model and adapt it to different instances and scenarios, the nearest-neighbor upsampling method was replaced with the lightweight universal upsampling operator (CARAFE).

Extensive experiments were conducted on a bearing defect dataset that we produced, and the results demonstrated that the detection accuracy of the defect detection method proposed in this paper reached 97.3%, with an overall average precision improvement of 1.5%, especially for dents and black spot defects improved by 2.2% and 3.9%, respectively, and that the detection speed can reach 100 FPS. An ablation experiment demonstrated the effectiveness of the proposed improvements, and a comparison with other algorithms also demonstrated the superiority of the improved method, meeting industrial inspection requirements. On a fabric dataset, the proposed method also showed improvement, with the mAP of 99%.

The YOLO series of network architectures has good openness, making it easy to introduce new network architectures and modules. In future research, further network optimization can be carried out for fine cracks on bearing surfaces, as well as interferences such as dust and oil stains, to improve the performance of the model.

## Figures and Tables

**Table 1:** System equipment model.

Equipment	Type
Camera	MV-CE200-10GM
Camera lens	MVL-KF5028M-12MP DH230-WTLN23
Light source	LTS-3DM260-R/W LTS-3DM260-R/W
PLC	FX3GA-40MR-CM

**Table 2:** Extended defect dataset.

	Helical Marks	Forging Waste	Black Spots	Dents	Scratches
Expansion	1140	1085	1148	1120	1167

**Table 3:** Bearing training, validation, and test set statistics.

	Training	Validation	Test	Total
Helical marks	747	197	196	1140
Forging Waste	721	182	182	1085
Black Spots	761	193	194	1148
Dents	753	184	183	1120
Scratches	783	192	192	1167

**Table 4:** Hardware environment and software version.

	Configuration
Hardware	Operating System: Linux Ubuntu
	CPU: Intel(R) Xeon(R) Platinum 8358P
	RAM: 30 G
	GPU: RTX A5000
Software	Python 3.8.13 + Pytorch 1.10 + CUDA11.1

**Table 5:** Network training parameters.

Training Parameter	Value
Batch Size	32
Dynamic Parameters	0.937
Learning Rate	0.01
Cosine Annealing Learning Rate	0.1
Data Augmentation	1.0
Input Size	640 × 640
Epochs	100

**Table 6:** Confusion matrix.

	Prediction	Positive	Negative
Real			
True		TP	FN
False		FP	TN

**Table 7:** Parameter adjustment and results.

Number	Image Size	Epochs	Batch Size	Learning Rate	Optimizer	mAP
exp1	640	100	32	0.01	SGD	97.3%
exp2	320	100	32	0.01	SGD	95.2%
exp3	640	100	16	0.01	SGD	96.5%
exp4	640	100	32	0.1	SGD	96.0%
exp5	640	100	32	0.01	Adam	90.4%
exp6	640	100	8	0.01	Adam	89.9%
exp7	640	100	16	0.1	SGD	95.6%
exp8	640	100	8	0.01	SGD	96.1%
exp9	640	100	64	0.01	SGD	96.1%

**Table 8:** Results of ablation experiments.

	C2f	CARAFE	SPD	mAP	FPS	Parameters
YOLOV5S				95.8%	106	7,023,610
C2F	✓			96.5%	111	6,932,122
CARAFE		✓		96.2%	98	7,157,666
SPD			✓	96.5%	105	8,693,666
C2F + CARAFE	✓	✓		96.9%	103	7,073,610
C2F + SPD	✓		✓	96.6%	111	8,559,610
CARAFE + SPD		✓	✓	96.7%	101	9,604,770
OURS	✓	✓	✓	97.3%	100	9,470,714

**Table 9:** Comparison of related methods on the bearing dataset.

	Helical Marks	Forging Waste	Black Spots	Dents	Scratches	mAP	FPS
SSD	94.3%	93.5%	92.8%	76.6%	91.8%	89.8%	71
Faster-RCNN	78.5%	93.6%	86.5%	69.2%	90.2%	83.6%	30
YOLOv3	97.5%	88.3%	89.8%	94.8%	87.7%	91.6%	110
YOLOv5	99.5%	96.2%	92.8%	96.7%	93.9%	95.8%	106
YOLOv6n	97.4%	93.8%	90.9%	94.3%	94.6%	94.2%	120
Yolov7	98.1%	89.5%	88.7%	92.3%	87.1%	91.1%	128
Ours	99.4%	96.5%	96.7%	98.9%	95.0%	97.3%	100

**Table 10:** Comparison of related methods on the cloth dataset.

	Hole	L_line	S_line	mAP	FPS
yolov3	99.5%	96.0%	97.5%	97.7%	116
yolov3-tiny	99.3%	81.5%	97.5%	92.8%	400
yolov5s	99.3%	97.9%	98.9%	98.3%	149
yolov6n	98.0%	95.1%	95.1%	96.3%	124
yolov7-tiny	98.7%	94.6%	98.2%	97.2%	164
yolov7	99.5%	90.8%	90.3%	93.5%	120
our	99.5%	98.2%	99.4%	99.0%	124

## Data Availability

The datasets generated during the current study are available from the corresponding author on reasonable request.
